# GWAS reveals loci associated with velopharyngeal dysfunction

**DOI:** 10.1038/s41598-018-26880-w

**Published:** 2018-05-31

**Authors:** Jonathan Chernus, Jasmien Roosenboom, Matthew Ford, Myoung Keun Lee, Beth Emanuele, Joel Anderton, Jacqueline T. Hecht, Carmencita Padilla, Frederic W. B. Deleyiannis, Carmen J. Buxo, Eleanor Feingold, Elizabeth J. Leslie, John R. Shaffer, Seth M. Weinberg, Mary L. Marazita

**Affiliations:** 1 Department of Human Genetics, Graduate School of Public Health, University of Pittsburgh, Pittsburgh, PA 15261 USA; 2 Center for Craniofacial and Dental Genetics, Department of Oral Biology, School of Dental Medicine, University of Pittsburgh, Pittsburgh, PA 15219 USA; 3 Cleft Craniofacial Center, Children’s Hospital of Pittsburgh, Pittsburgh, PA USA; 4 Department of Pediatrics, McGovern Medical School and School of Dentistry UT Health at Houston, Houston, TX 77030 USA; 5 Department of Pediatrics, College of Medicine; and Institute of Human Genetics, National Institutes of Health; University of the Philippines Manila, Manila, 1101 The Philippines; 6 Department of Surgery, Plastic and Reconstructive Surgery, University of Colorado School of Medicine, Denver, CO 80045 USA; 7 Dental and Craniofacial Genomics Core, School of Dental Medicine, University of Puerto Rico, San Juan, 00936 Puerto Rico; 8 Department of Human Genetics, Emory University School of Medicine, Emory University, Atlanta, GA 30322 USA

## Abstract

Velopharyngeal dysfunction (VPD) occurs when the muscular soft palate (velum) and lateral pharyngeal walls are physically unable to separate the oral and nasal cavities during speech production leading to hypernasality and abnormal speech reduction. Because VPD is often associated with overt or submucous cleft palate, it could be present as a subclinical phenotype in families with a history of orofacial clefting. A key assumption to this model is that the overt and subclinical manifestations of the orofacial cleft phenotype exist on a continuum and therefore share common etiological factors. We performed a genome-wide association study in 976 unaffected relatives of isolated CP probands, 54 of whom had VPD. Five loci were significantly (p < 5 × 10^−8^) associated with VPD: 3q29, 9p21.1, 12q21.31, 16p12.3 and 16p13.3. An additional 15 loci showing suggestive evidence of association with VPD were observed. Several genes known to be involved in orofacial clefting and craniofacial development are located in these regions, such as *TFRC*, *PCYT1A*, *BNC2* and *FREM1*. Although further research is necessary, this could be an indication for a potential shared genetic architecture between VPD and cleft palate, and supporting the hypothesis that VPD is a subclinical phenotype of orofacial clefting.

## Introduction

Velopharyngeal dysfunction (VPD) refers to an inability to close the opening between the nasal cavity and the oral cavity during speech. This occurs because the muscular soft palate (velum) and lateral pharyngeal walls are physically unable to make a sufficient seal of the oral cavity from the nasal cavity during speech production. As a consequence, air tends to escape into the nasal cavity during speech, resulting in hypernasality and excess air emissions. The causes of VPD are heterogeneous and are the basis of three subtypes of VPD: velopharyngeal incompetency (caused by a lack of neuromotor competency), velopharyngeal mislearning (caused by maladaptive articulatory habits) and velopharyngeal insufficiency (VPI, caused by insufficient tissue or mechanical restriction)^1,2^. For example, a congenitally short palate and/or deep nasopharynx can alter the geometry of the velopharyngeal apparatus, such that the soft palate is no longer able to effectively create a seal against the posterior wall of the nasopharynx^2,3^. Although VPD can occur as the result of surgical procedures, such as adenoidectomies, the most common congenital cause of VPD is cleft palate (CP) or submucous cleft palate (smCP), which can occur as isolated malformations or as part of a syndrome^4^. After primary palatal repair surgeries, approximately 30% of CP patients require additional surgery for VPD^1^.

VPD can also occur in the absence of an overt orofacial cleft^5^, and some cases have been reported with autosomal-dominant inheritance^6,7^. Detailed phenotyping in these “isolated” VPD cases shows that these result from structural deficiencies in the anatomical components that comprise the velopharyngeal mechanism. The genetic basis of isolated VPD is poorly understood and the autosomal-dominant families have yet to be genetically mapped. However, given the association with CP and the structural deficiencies of the palate, genes and pathways implicated in the pathogenesis of secondary palate clefting may provide some clues^8,9^.

The purpose of the current study is to examine the influence of common genetic variants on VPD. To accomplish this, we performed a genome-wide association study (GWAS) on a sample of unaffected relatives from families with a history of CP or smCP, who had been assessed for VPD.

## Materials and Methods

### Participants

Our study sample consisted of 976 relatives within three degrees of relatedness of probands with an isolated CP (437 male, 539 female; mean age = 29.7 ± 16.29) and who were not affected with an overt CP by both self-report and in-person assessment of their cleft status. The participants were recruited as part of the larger Pittsburgh Orofacial Cleft Study^10^ at different US and international sites: Pittsburgh (n = 281), St. Louis (n = 51), Texas (n = 299), Colorado (n = 39), Hungary (n = 99), Colombia (n = 16), Philippines (n = 171), and Puerto Rico (n = 20).

### Speech assessment

Structured and spontaneous speech samples were recorded for all participants using a Canon 7D camera (Canon USA, Melville, NY). The structured speech paragraphs in English, Spanish and Tagalog can be found in the online supplemental material. Medical and surgical history was documented for all subjects, with a particular focus on speech pathology and palatal surgery. Using the Pittsburgh Weighted Speech Scale^11^, the speech samples were rated for the presence of VPD by an experienced speech and language pathologist (MF). A speech score was given based on the presence of audible nasal emission and nasal turbulence, nasality, phonation and articulation patterns. Subjects with a score higher than three (which is the cut-off for clinical significance) were considered to have VPD. Using this threshold, a total of 54 participants (20 males, 34 females) were diagnosed with VPD.

### Genotyping, quality control, population structure and imputation

DNA was extracted from saliva or blood, and genotyped for 541787 SNPs on an Illumina HumanCore + Exome array plus 15890 SNPs of custom content covering candidate genes for overt clefts. Genetic data cleaning and quality control analyses were performed as described previously^12^. In brief, samples were interrogated for genetic sex, chromosomal aberrations, relatedness, genotype call rate, and batch effects. SNPs were interrogated for call rate, discordance among 72 duplicate samples, Mendelian errors among HapMap controls (parent-offspring trios), deviations from Hardy-Weinberg equilibrium, and sex differences in allele frequencies and heterozygosity. Filters applied to genotyped SNPs are described in Supplementary Table S1.

Imputation of non-genotyped variants was performed via IMPUTE2^13^, using haplotypes from the 1000 Genomes Project Phase 3 as the reference. We converted imputed probabilities to most-likely genotypes using a genotype probability threshold of 0.9. We then filtered out imputed SNPs with an info score of <0.5. Masked variant analysis, in which genotyped SNPs were imputed in order to assess imputation quality, indicated high accuracy of imputation. Genetic association with VPD was tested for genotyped and imputed SNPs with MAF >5% and which did not show evidence of extreme deviation for the Hardy-Weinberg equilibrium.

### Association analysis

Genetic association with VPD was tested for SNPs with MAF >5% using a mixed-models approach as implemented in EMMAX^14^, which explicitly models the variance due to the kinship (comprising both the family relatedness and population structure) in the sample. 54 unaffected relatives of patients with CP were diagnosed with VPD and compared to 922 unaffected relatives of patients with CP, who are not showing VPD. Sex, age, age^2^, and site (as a proxy for language) were included as covariates. Principal components of ancestry were not included because variation due to population structure was already explicitly modeled by the kinship matrix. Autosomal SNP genotypes were modeled additively. For SNPs on the X-chromosome, genotypes were coded as 0, 1, and 2 for females, and were coded as 0 or 2 for males in order to maintain the same scale between sexes. The conventional Bonferroni-corrected threshold of 5 × 10^−8^ was set for genome-wide statistical significance; 5 × 10^−6^ was the threshold for suggestive hits.

### Functional annotation

Potential genes of interest were identified based on physical proximity of ±500 kb from the lead SNP at each genome-wide significant locus. These genes were queried in the following online databases: The Mouse Genome Informatics (MGI) database^15^, which was used to annotate expression in relevant tissues and phenotypic consequences, the VISTA enhancer database^16^, which was used to annotate active enhancer elements in relevant tissues, and OMIM and PubMed, which were used to annotate human phenotypic information. The following genes were considered to be of interest during annotation: genes involved in orofacial clefting, in speech pathology caused by structural and central differences, and in craniofacial development.

### Data availability

SNPs used in the current study are available in the dbGAP repository (https://www.ncbi.nlm.nih.gov/gap; accession number phs000774.v1.p1).

### Compliance with Ethical Standards

Written informed consent was obtained from all individual participants in the study. All procedures performed in studies involving human participants were in accordance with the ethical standards of the institutional research committees and with the 1964 Helsinki declaration and its later amendments or comparable ethical standards: University of Pittsburgh IRB #PRO09060553 and IRB0405013 (covering both the Pittsburgh and Hungary sites); UT Health Committee for the Protection of Human Subjects #HSC-DB-09-0508 and #HSC-MS-03-090; Colorado Multiple Institutional Review Board #10-0055, Washington University in St. Louis HRPO #03-087, University of the Philippines Manilla, IRB00002908, FWA00018728; University of Puerto Rico Medical Sciences Campus IRB, IRB protocol 0640111 l. The “Fundacion Clinica Noel de Medellin” IRB approved the study protocol for the subjects in Colombia, an IRB approval number is not available for this site.

## Results

### Genetic association

**Figure 1:**
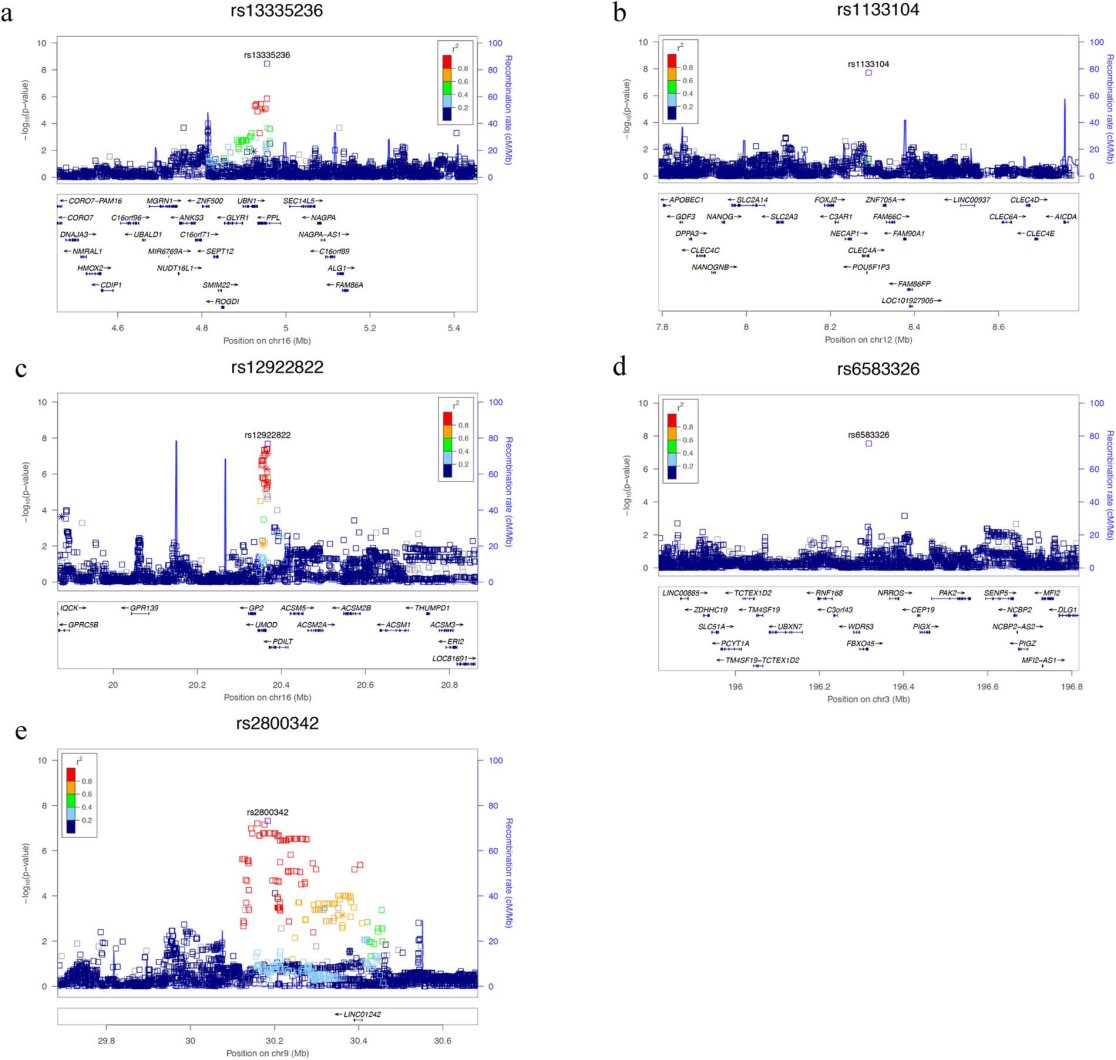
(**a**–**e**) LocusZoom plots of genome-wide significant (p < 5*10E-08) associations. LocusZoom plots show the association (left y-axis; log10-transformed p-values). Genotype SNPs are represented by stars, imputed SNPs are represented by squares. Shading of the points represent the linkage disequilibrium (r2, based on the 1000 Genomes Project) between each SNP and the top SNP. The blue overlay shows the recombination rate (right y-axis). Positions of genes are shown below the plot.

Our GWAS revealed five genome-wide significant associations at loci 3q29 (lead SNP: rs6583326, p = 2.86 × 10^−8^), 9p21.1 (lead SNP: rs2800342, p = 4.88 × 10^−8^), 12q21.31 (lead SNP: rs1133104, p = 1.96 × 10^−8^), 16p12.3 (lead SNP: rs12922822, p = 2.08 × 10^−8^) and 16p13.3 (lead SNP: rs13335236, p = 3.50 × 10^−9^) (Table 1). Two of these SNPs were intragenic: SNP rs1133104 (12q21) is located in the last exon of *CLEC4A* and rs13335236 (16p13) is located intronic in *PPL*. The LocusZoom plots^17^ of these results are shown in Fig. 1, and the Manhattan and QQ plots are shown in Supplementary Figure S1. In addition, 15 loci showed a suggestive association (p < 5 × 10^−6^) with VPD (Table 1). LocusZoom plots of all suggestive associations are displayed in Supplementary Figure S2 and results are described in Supplementary Table S2.

**Table 1:** Genome-wide significant (p < 5*10E-08) and suggestive (p < 5*10E-06) associations with VPD.

Locus	SNP	Chr	BP position	P	A1	A2	MAF	Annotated genes
3p24.3	rs13095954	3	18108126	1.05E-07	G	C	0.07358	*SATB1*
3q29	rs6583326	3	196317308	2.86E-08	T	C	0.1434	*TFRC, PCYT1A*
4q22.3	rs11727234	4	96807975	6.95E-07	G	T	0.05155	—
5p13.1	rs56276612	5	40427385	1.19E-07	T	G	0.09129	—
6p12.2	rs10948810	6	54176227	4.08E-07	C	T	0.05169	—
6q26	rs3900809	6	161135202	2.94E-07	G	A	0.05169	*MAP3K4, PARK2*
8q21.2	rs34927688	8	8622794	7.59E-07	G	A	0.07439	—
9p21.1	rs2800342	9	30183553	4.88E-08	T	A	0.05983	—
9p22.2	rs1538101	9	16710870	3.39E-07	G	A	0.05169	*BNC2*
9p22.3	rs72702916	9	14484998	1.47E-07	C	G	0.07164	*FREM1*
11q25	rs56160206	11	133921939	8.02E-08	CA	C	0.1263	*GLB1L2, B3GAT1*
12q21.31	rs1133104	12	8291122	1.96E-08	G	T	0.1712	—
14q13.1	rs73257280	14	32270651	6.48E-07	C	CT	0.05113	—
14q23.3	chr14:66527789	14	66527789	4.48E-07	T	A	0.05236	—
16p12.1	rs77085399	16	22002367	9.89E-07	C	T	0.16	—
16p12.3	rs12922822	16	20367645	2.08E-08	T	C	0.1498	—
16p13.3	rs13335236	16	4955256	3.50E-09	C	T	0.05733	*NAGPA, RBFOX1*
18q21.32	rs11877326	18	56503556	2.64E-07	A	G	0.07174	—
18q22.1	rs66549549	18	62628423	4.39E-07	T	G	0.05215	—
22q12.1	rs9613645	22	28947402	7.57E-08	T	A	0.08383	*TTC28, KREMEN1*

Several possible biologically relevant candidate genes (e.g., *PCYT1A, FREM1)* were located at the five genome-wide significant loci. To ensure a more comprehensive evaluation, genes within 500 kb of each lead SNP were queried for possible roles in orofacial clefting, speech development, and/or development of the nasopharyngeal region. Corroborating evidence, such as expression in relevant tissues or putative roles in relevant human syndromes, was found for eight of the 20 loci as discussed below.

## Discussion

This GWAS of VPD identified five genome-wide significant associations and 15 suggestive associations. We were not able to replicate these results, because, to our best knowledge, no suitable replication cohort is available. Although the genetic basis of VPD is largely unknown, several of these loci were located near potentially relevant candidate genes, including some previously implicated in orofacial clefting. *PCYT1A*, located 350 kb upstream of the lead SNP at the 3q29 locus, has been shown to be associated with increased risk for NSCL/P, through an epistatic interaction with *BHMT*^18^. Furthermore, a microdeletion in this locus is furthermore associated with a delayed development, especially in speech^19^. The association at this locus, however, is based on a single imputed SNP. We also observed borderline associations with several variants in *TTC28* (locus 22q12.1). Conte and colleagues found an association between copy number variants in *TTC28* and orofacial development^20^. A microdeletion in the same genetic region was found in a child with Pierre-Robin Sequence (including cleft palate) and Neurofibromatosis type 2^21^. Moreover, the *ttc28* mouse mutant shows cranial abnormalities with abnormal maxillary morphology, further suggesting the potential for this gene to impact palatogenesis.

Several other associated loci contained candidate genes known to be involved more generally with craniofacial development. For instance, *FREM1* (near locus 9p22.3) has been shown to play a role in the fusion of the nasal processes during gestation^22^, and was implicated in human upper lip morphology in a recent GWAS of facial shape^23^. In humans, mutations in this gene result in several Mendelian conditions affecting the midline facial structures, such as BNAR syndrome (OMIM #608980) and trigonocephaly (OMIM #190440)^22,24^. Trigonocephaly is in 34% of the cases associated with speech and/or language delay^25^. *KREMEN1* (near locus 22q12.2) is a modulator of *WNT* signaling, crucial for neural tube closure^26^. *TFRC*, located 500 kb from the lead SNP of locus 3q29, is involved in craniofacial morphogenesis by regulating *TGFß* and *BMP* signaling activation^27^.

Interestingly, one of the identified loci contained genes involved in the neural control of speech production. *NAGPA* at locus 16p13.3 is involved in stuttering^28^ and focal epilepsy with speech disorders^29^, respectively. Neurophysiological dysfunction is a known cause of VPD^4^, so it is possible that variants in these genes might influence the movement of the muscles comprising the soft palate.

We have hypothesized that isolated VPD may represent a subclinical phenotype in families with a history of orofacial clefting^10^. In the context of orofacial clefting, subclinical phenotypes can be conceptualized as incomplete (or intermediate) expressions of the risk factors for the overt defect or as pleiotropic expressions in related tissues/structures. Such subclinical phenotypes have now been extensively documented in the clinically unaffected relatives of affected individuals within families affected with orofacial clefts^10,30^. A key assumption is that the overt and subclinical manifestations of the orofacial cleft phenotype share common etiological factors. If this model is correct, then at least some of the genes that underlie orofacial cleft susceptibility, particularly those involved in clefts of the secondary palate, may also underlie VPD.

If the hypothesis is correct that CP and VPD (partially) share their genetic etiology, it may be valuable to investigate the effect of genes known to be involved in the etiology of CP, in subjects with VPD. However, our understanding of the genetic basis of isolated CP is largely incomplete. Associations between CP and SNPs in *FAF1*^31^, *FOXE1*^32^, and *GRHL3*^9,33^ have been described. No variants in or near these genes were associated with VPD in our study cohort. This does not preclude the possibility that additional (yet to be identified) variants involved in CP will contribute to VPD and vice versa. Unlike overt CP, smCP is usually not immediately diagnosed at birth. A recent candidate gene study of six SNPs in loci strongly associated with orofacial clefting, did not show any association between these SNPs and smCP^34^, leaving the genetic basis of smCP unknown. Since there is a high prevalence of VPD in patients with smCP, the genetic loci identified in this study are also good candidate genes for both overt CP and smCP.

This study represents the first attempt to identify genetic variants associated with VPD. Although we observed several promising associations, we are not currently able to independently replicate any of these signals due to a lack of additional datasets. Our study was also limited by a lack of objective assessments of VPD, such as acoustic nasalence data during speech assessed by nasometry, or visualization of the velopharyngeal mechanism during speech assessed by video nasopharyngeal endoscopy. Including these types of assessments may yield additional insights into the genetic basis of VPD. Furthermore, the presence of smCP could not be determined in this dataset. Thus, it is possible that the presence of VPD is actually due to undiagnosed smCP. Although we hypothesize that VPD may be a subclinical phenotype of CP, we did not find strong evidence of association between the signals identified in this study and CP.

## Electronic supplementary material

Supplemental Material

Dataset 1
