# Supplementary material for: GWAS reveals loci associated with velopharyngeal dysfunction
Source: Sci Rep. 2018 May 31;8:8470. doi: 10.1038/s41598-018-26880-w (PMC5981322; doi:10.1038/s41598-018-26880-w)
Supplement: Supplementary file 1 — Supplemental Material [file 41598_2018_26880_MOESM1_ESM.pdf]

GWAS reveals loci associated with velopharyngeal dysfunction

Chernus J[1,$], Roosenboom J[2,$], Ford M[3], Lee MK[2], Emanuele B[2], Anderton J[2], Hecht JT[4], Padilla C[5], Deleyiannis FW[6], Buxo CJ[7], Feingold E[1], Leslie EJ[8], Shaffer JR[1,2], Weinberg SM[1,2*], Marazita ML[1,2*]

Supplementary text: Structured speech samples

Article: GWAS reveals loci associated with velopharyngeal dysfunction
Journal: Human Genetics
Author: Chernus J *et al*.
Corresponding author: Seth M Weinberg, Center for Craniofacial and Dental Genetics, University of Pittsburgh, USA. Smwst46@pitt.edu

English

You wished to know all about my grandfather. Well, he is nearly ninety-three years old; he dresses himself in an ancient black frock coat, usually minus several buttons, yet he still thinks as swiftly as ever. A long, flowing beard clings to his chin, giving those who observe him a pronounced feeling of the utmost respect. When he speaks, his voice is just a bit cracked and quivers a trifle. Twice each day he plays skillfully and with zest upon our small organ. Except in the winter when the ooze or snow or ice prevents, he slowly takes a short walk in the open air each day. We have often urged him to walk more and smoke less, but he always answers, “Banana oil!” Grandfather likes to be modern in his language.

Spanish

La oveja es un animal herbívoro. Se alimenta de hierba. Habita en todos los climas. Es un animal manso y resistente. Se mueve constantemente, pero es dócil a la voz del pastor y se deja guiar por los perros. Todo es útil en la oveja. La lana sirve para fabricar vestidos, mantas y alfombras. La piel se usa para abrigos y objetos de adorno. Su carne es sabrosa y con su leche se hacen quesos.

Tagalog

Ang Hardin. Sa tabi ng ilog ay may hardin, puno ng rosas, poinsetya, at sampaguita. Makulay ang bulaklak at mabango ang halimuyak. Gandang-ganda ako sa yabong ng mga halaman. Sa paligid nakakalat ang gumamela at dama de noche. Masarap amuyin ang mga bulaklak sa loob ng hardin. Nakagagaan din ng pakiramdam pagmasdan ang mga bulaklak. Maliwanag ang sikat ng araw sa hardin, kaya't ang mga halaman ay walang kupas ang mga kulay at bango. Madaling mahanap ang hardin na ito, dahil, sundan lang ang halimuyak at ang mga paru-paro.

VPI

Figures

Figure S1

Fig S1 Manhattanplot and QQ plot of the genome-wide association study.
Lamba=1.014

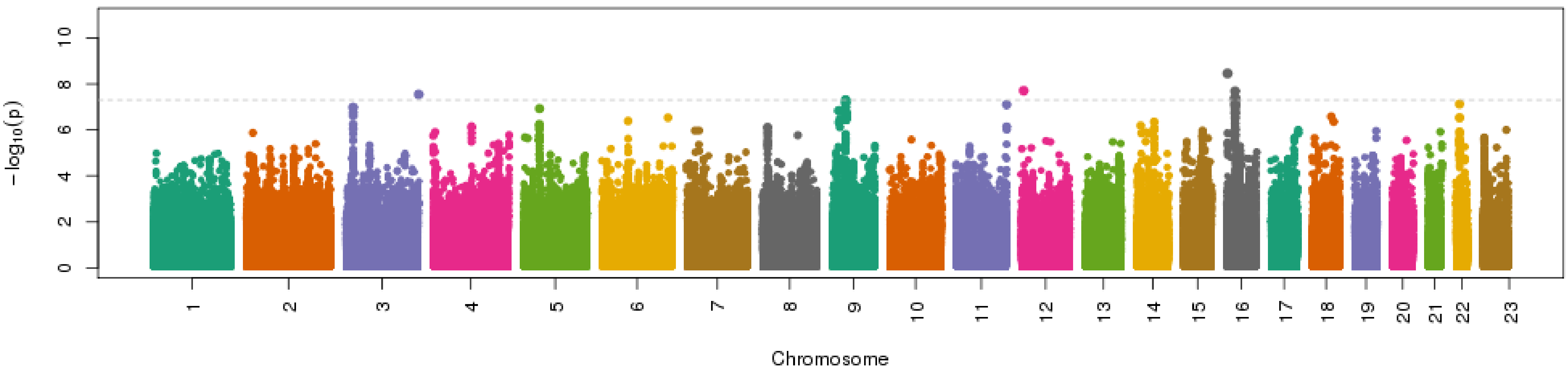
$-\log_{10}(p)$
Chromosome
1
2
3
4
5
6
7
8
9
10
11
12
13
14
15
16
17
18
19
20
21
22
23

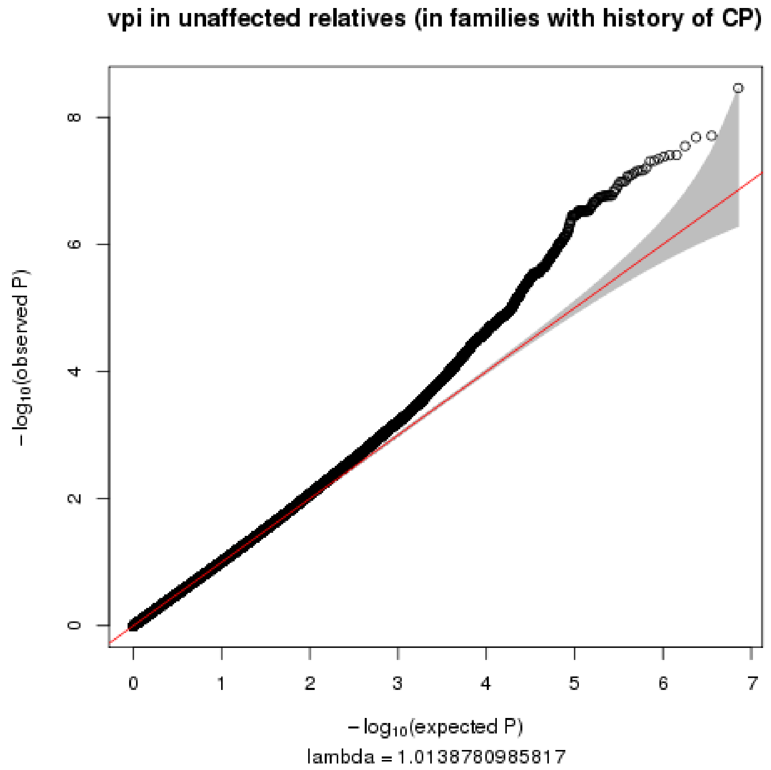
vpi in unaffected relatives (in families with history of CP)
$-\log_{10}$(observed P)
$-\log_{10}$(expected P)
lambda = 1.0138780985817

Figure S2

Fig S2 All LocusZoom plots of suggestive associations (p<5*10E-07). Genotype SNPs are represented by stars, imputed SNPs are represented by squares

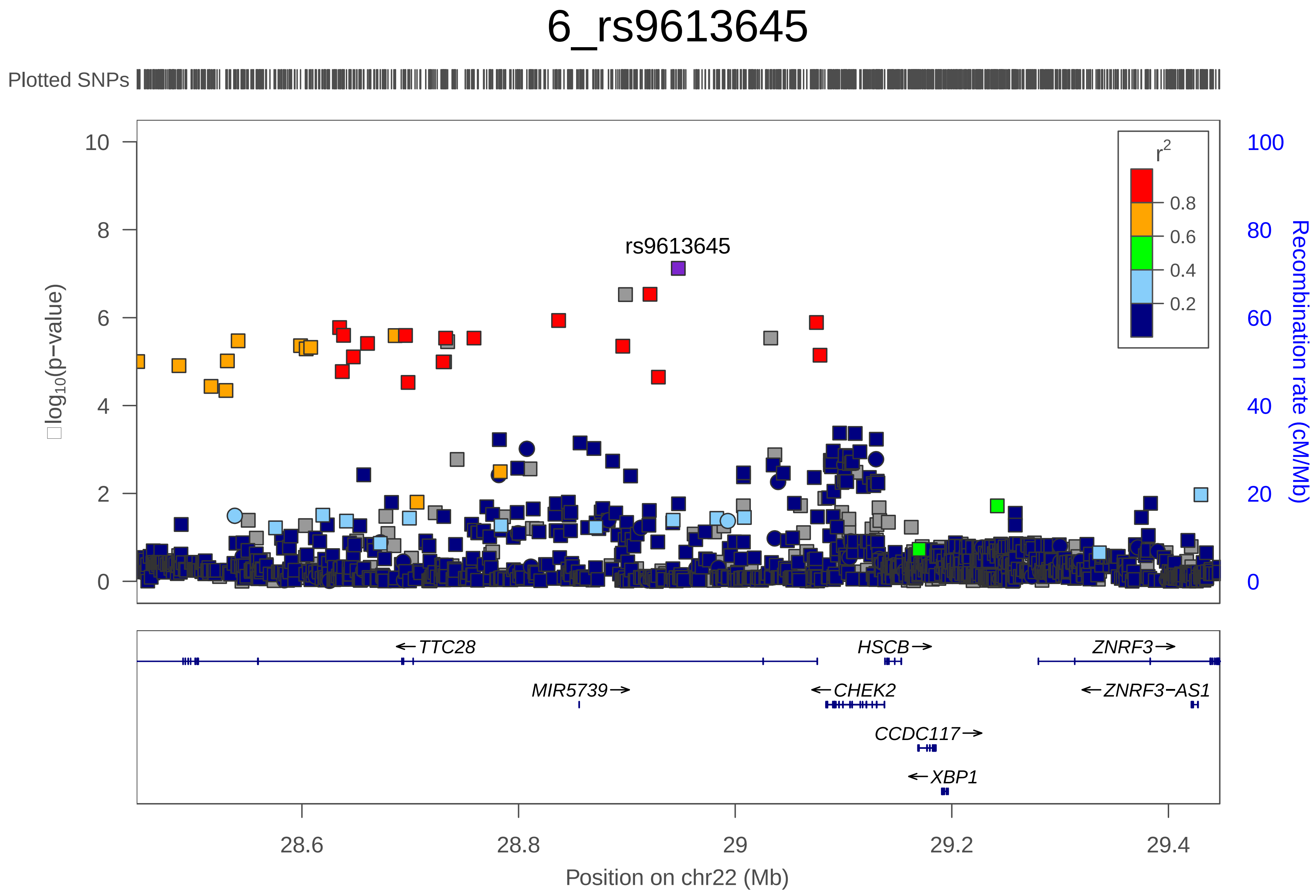
6_rs9613645
Plotted SNPs
−log10(p−value)
10
8
6
4
2
0
rs9613645
r2
0.8
0.6
0.4
0.2
100
80
60
40
20
0
Recombination rate (cM/Mb)
TTC28
HSCB
ZNRF3
MIR5739
CHEK2
ZNRF3−AS1
CCDC117
XBP1
28.6
28.8
29
29.2
29.4
Position on chr22 (Mb)

7_rs56160206

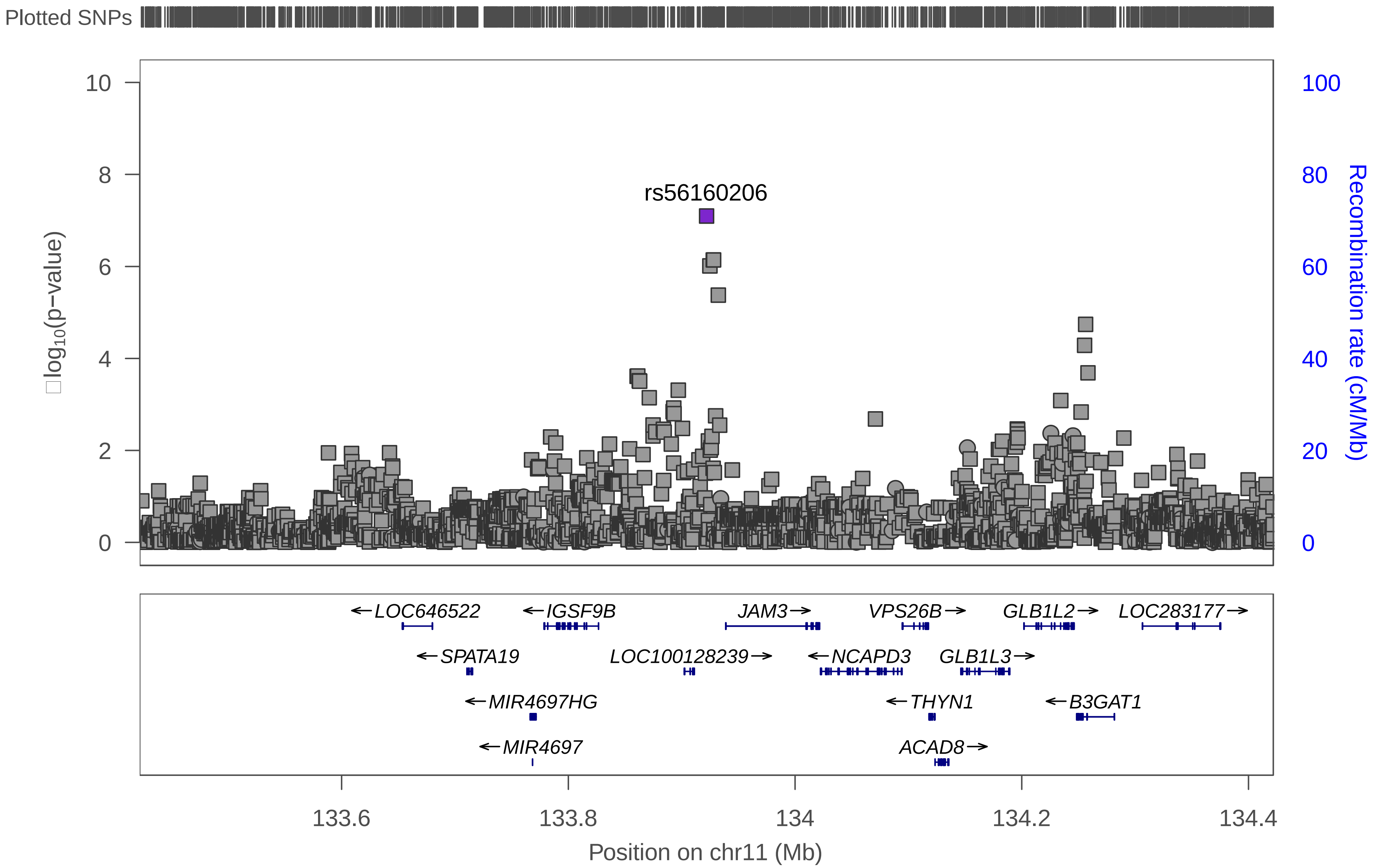

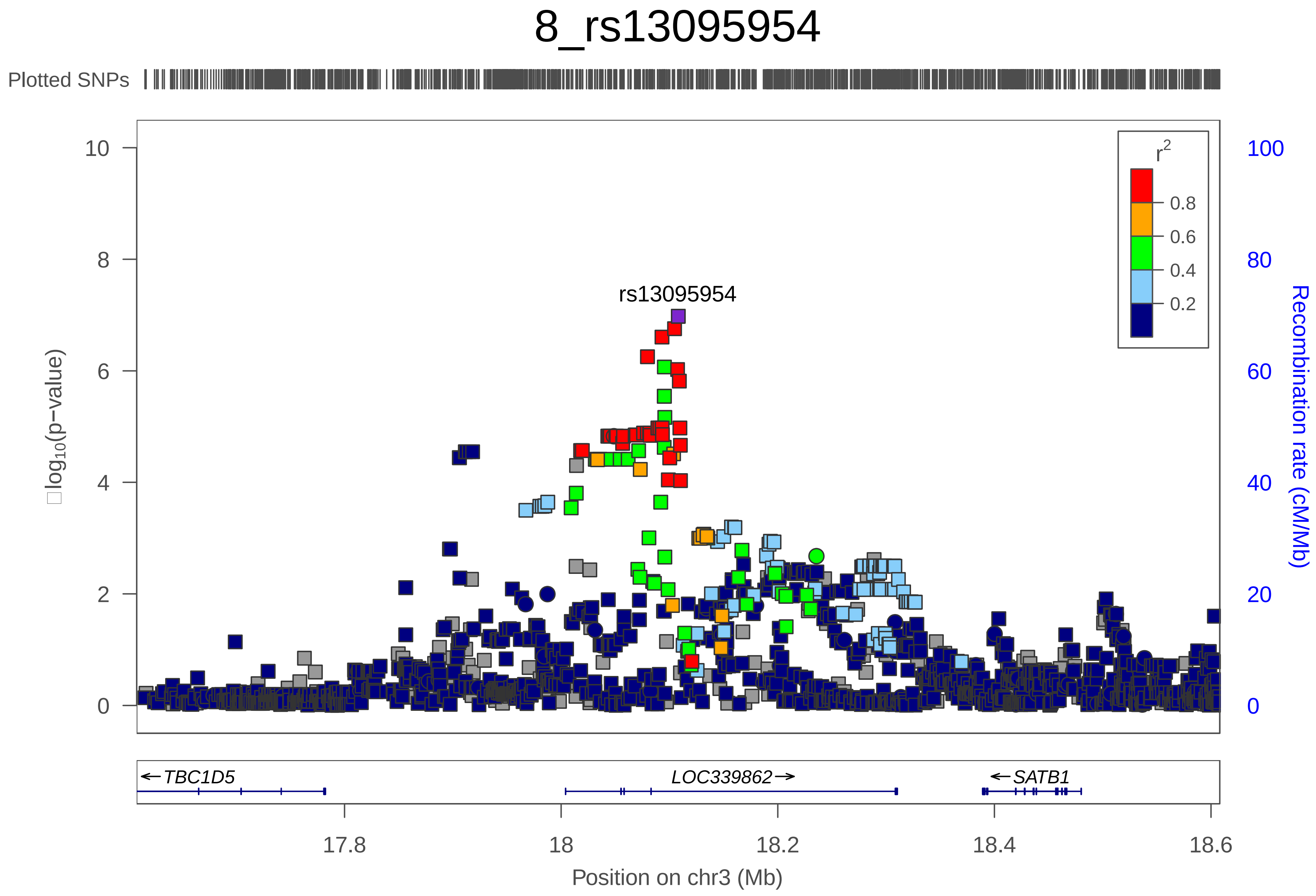
8_rs13095954
Plotted SNPs
rs13095954
r2
0.8
0.6
0.4
0.2
log10(p−value)
10
8
6
4
2
0
Recombination rate (cM/Mb)
100
80
60
40
20
0
TBC1D5
LOC339862
SATB1
17.8
18
18.2
18.4
18.6
Position on chr3 (Mb)

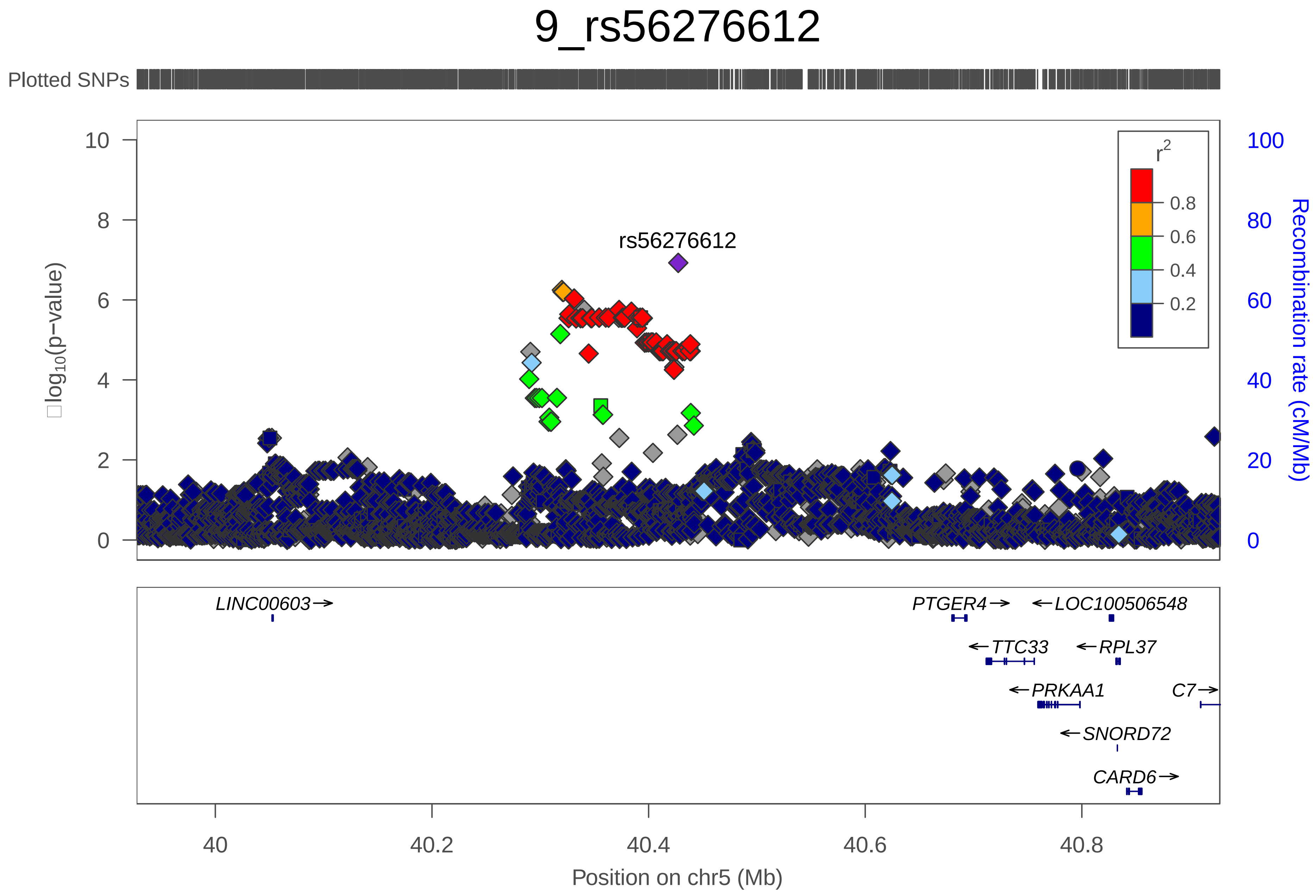
9_rs56276612
Plotted SNPs
rs56276612
log10(p−value)
Recombination rate (cM/Mb)
r2
0.8
0.6
0.4
0.2
10
8
6
4
2
0
100
80
60
40
20
0
LINC00603
PTGER4
LOC100506548
TTC33
RPL37
PRKAA1
C7
SNORD72
CARD6
40
40.2
40.4
40.6
40.8
Position on chr5 (Mb)

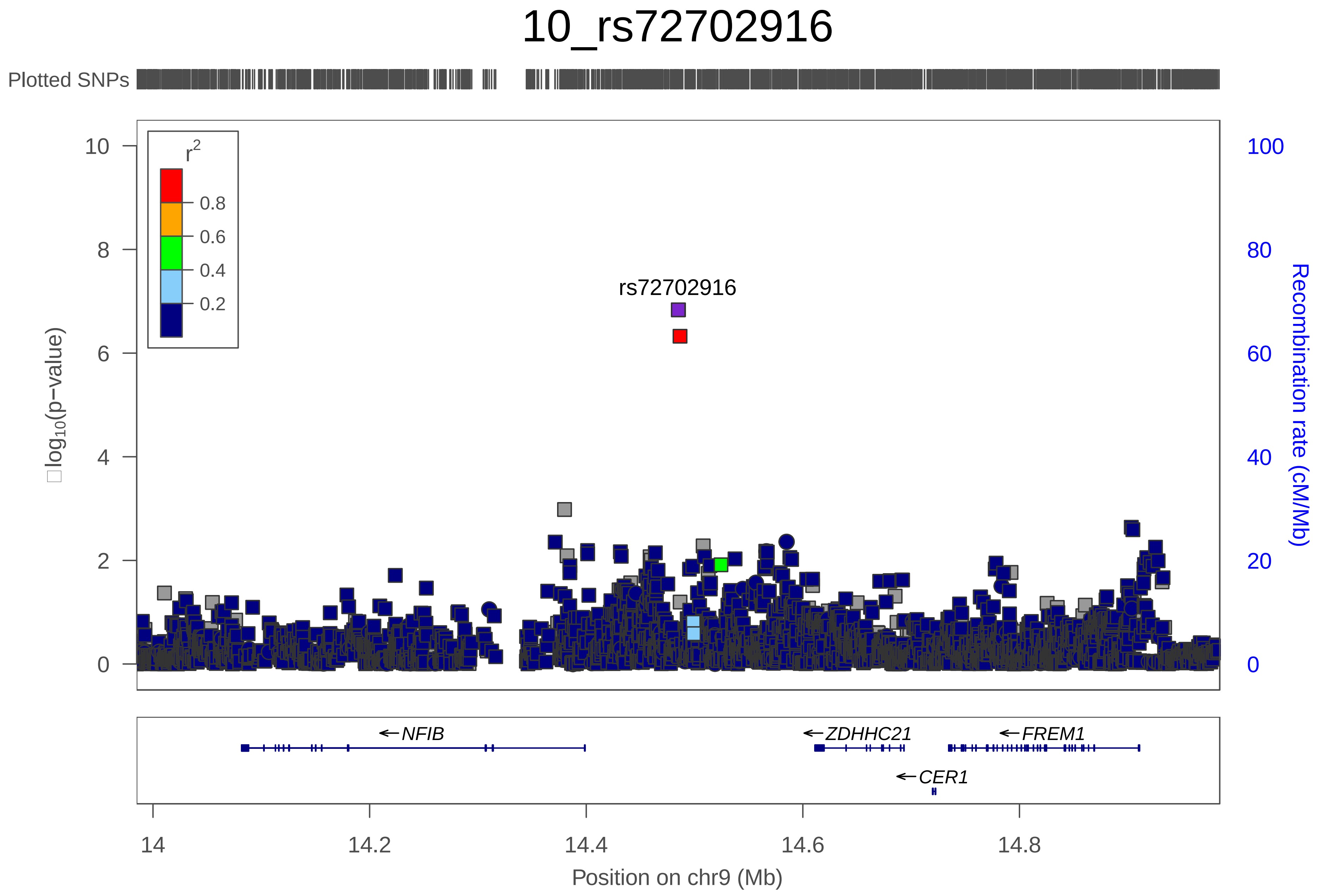

10_rs72702916
Plotted SNPs
r2
0.8
0.6
0.4
0.2
rs72702916
log10(p−value)
Recombination rate (cM/Mb)
NFIB
ZDHHC21
FREM1
CER1
Position on chr9 (Mb)

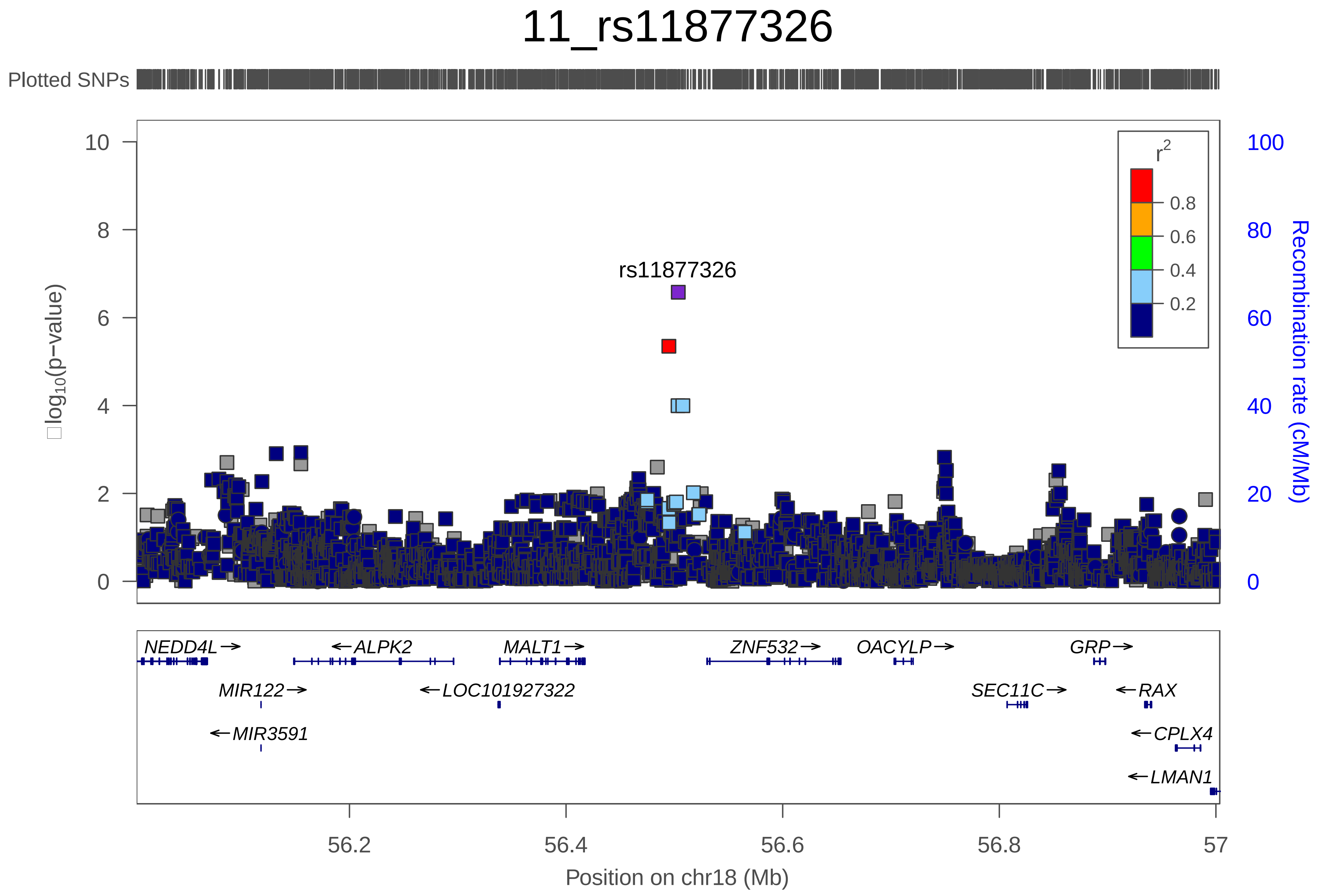
11_rs11877326
Plotted SNPs
rs11877326
r2
0.8
0.6
0.4
0.2
−log10(p−value)
Recombination rate (cM/Mb)
10
8
6
4
2
0
100
80
60
40
20
0
NEDD4L
ALPK2
MALT1
ZNF532
OACYLP
GRP
MIR122
LOC101927322
SEC11C
RAX
MIR3591
CPLX4
LMAN1
56.2
56.4
56.6
56.8
57
Position on chr18 (Mb)

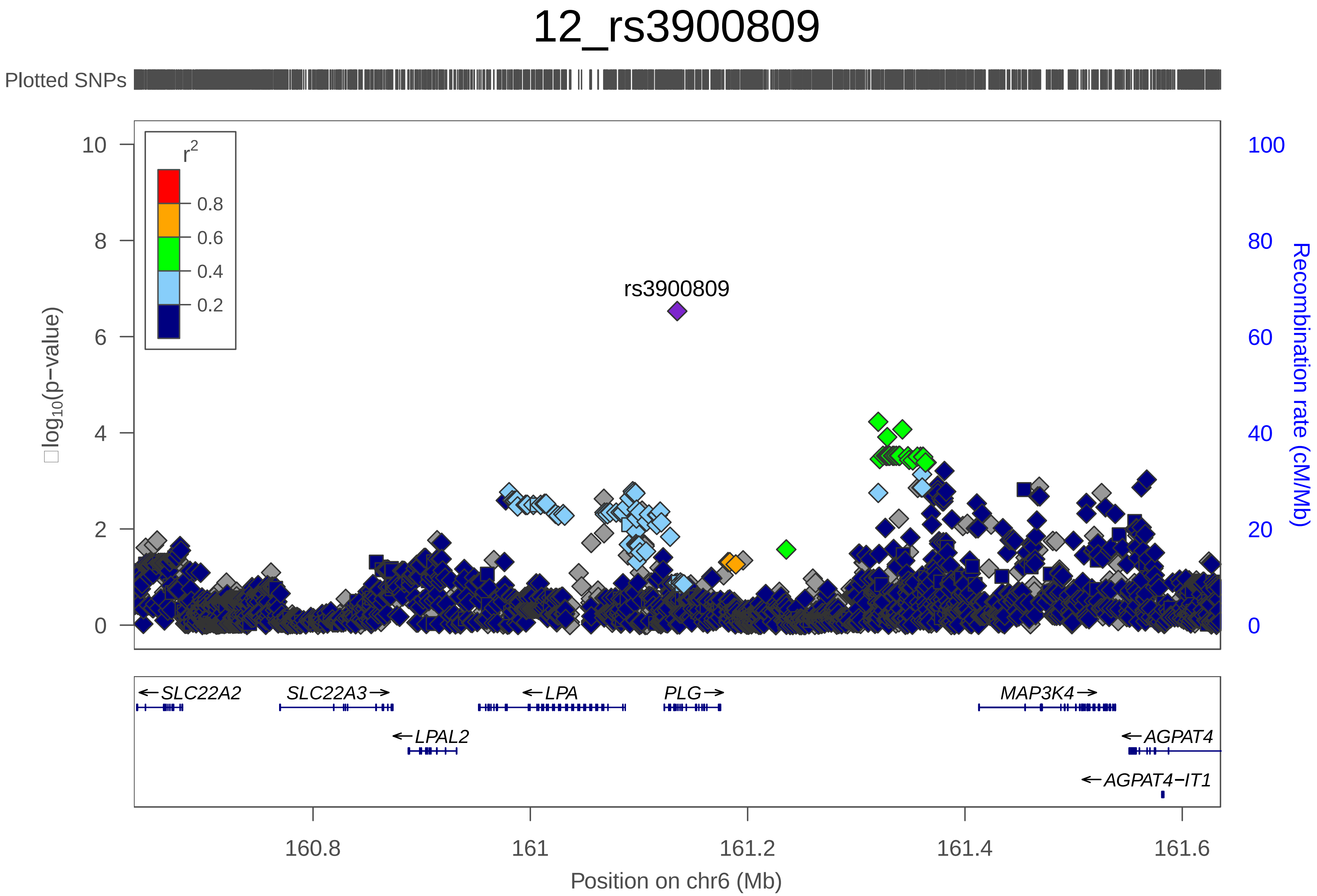
12_rs3900809
Plotted SNPs
r²
0.8
0.6
0.4
0.2
rs3900809
log10(p−value)
Recombination rate (cM/Mb)
10
8
6
4
2
0
100
80
60
40
20
0
SLC22A2
SLC22A3
LPA
PLG
MAP3K4
LPAL2
AGPAT4
AGPAT4−IT1
160.8
161
161.2
161.4
161.6
Position on chr6 (Mb)

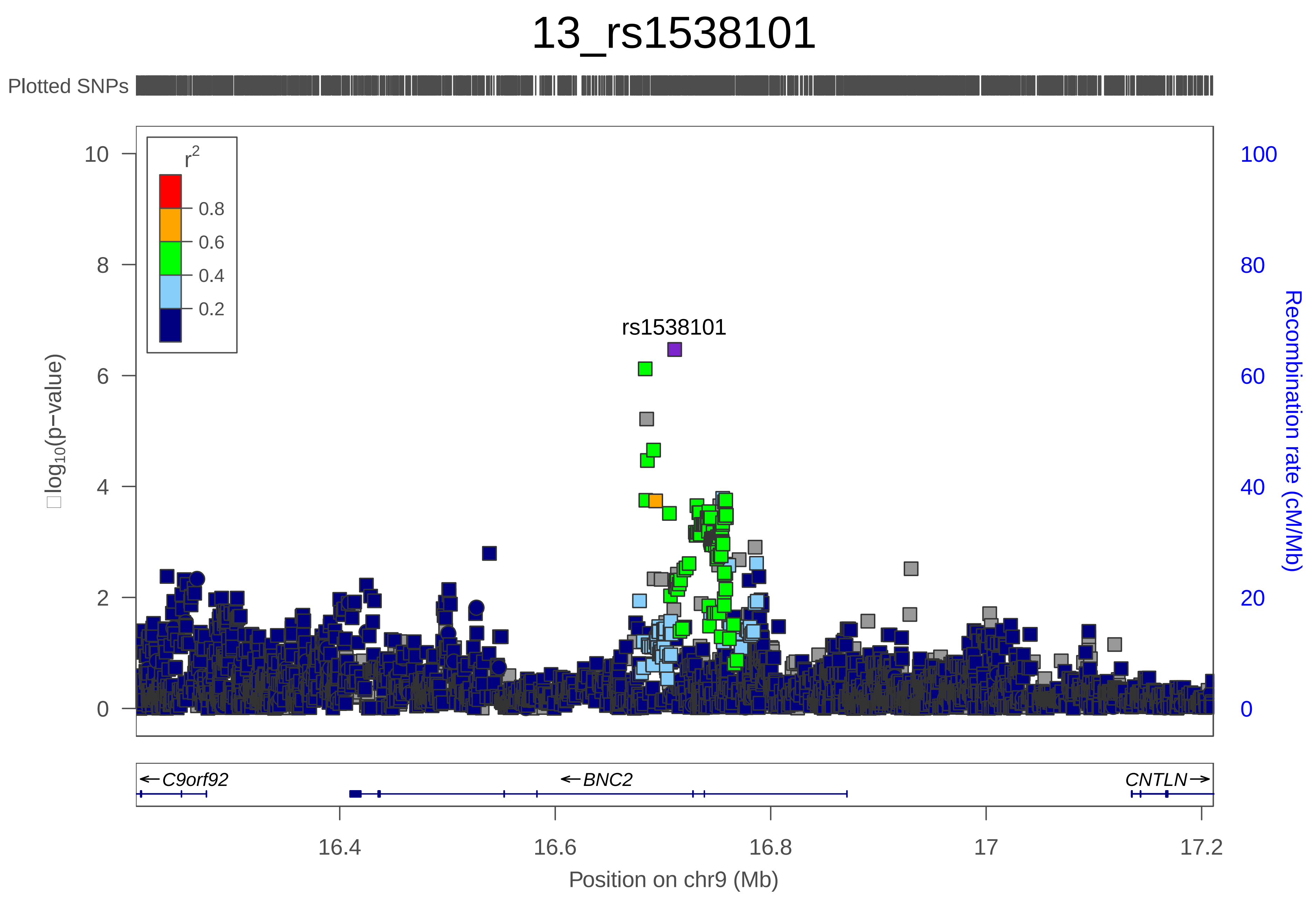

13_rs1538101
Plotted SNPs
r2
0.8
0.6
0.4
0.2
rs1538101
10
8
6
4
2
0
100
80
60
40
20
0
log10(p−value)
Recombination rate (cM/Mb)
C9orf92
BNC2
CNTLN
16.4
16.6
16.8
17
17.2
Position on chr9 (Mb)

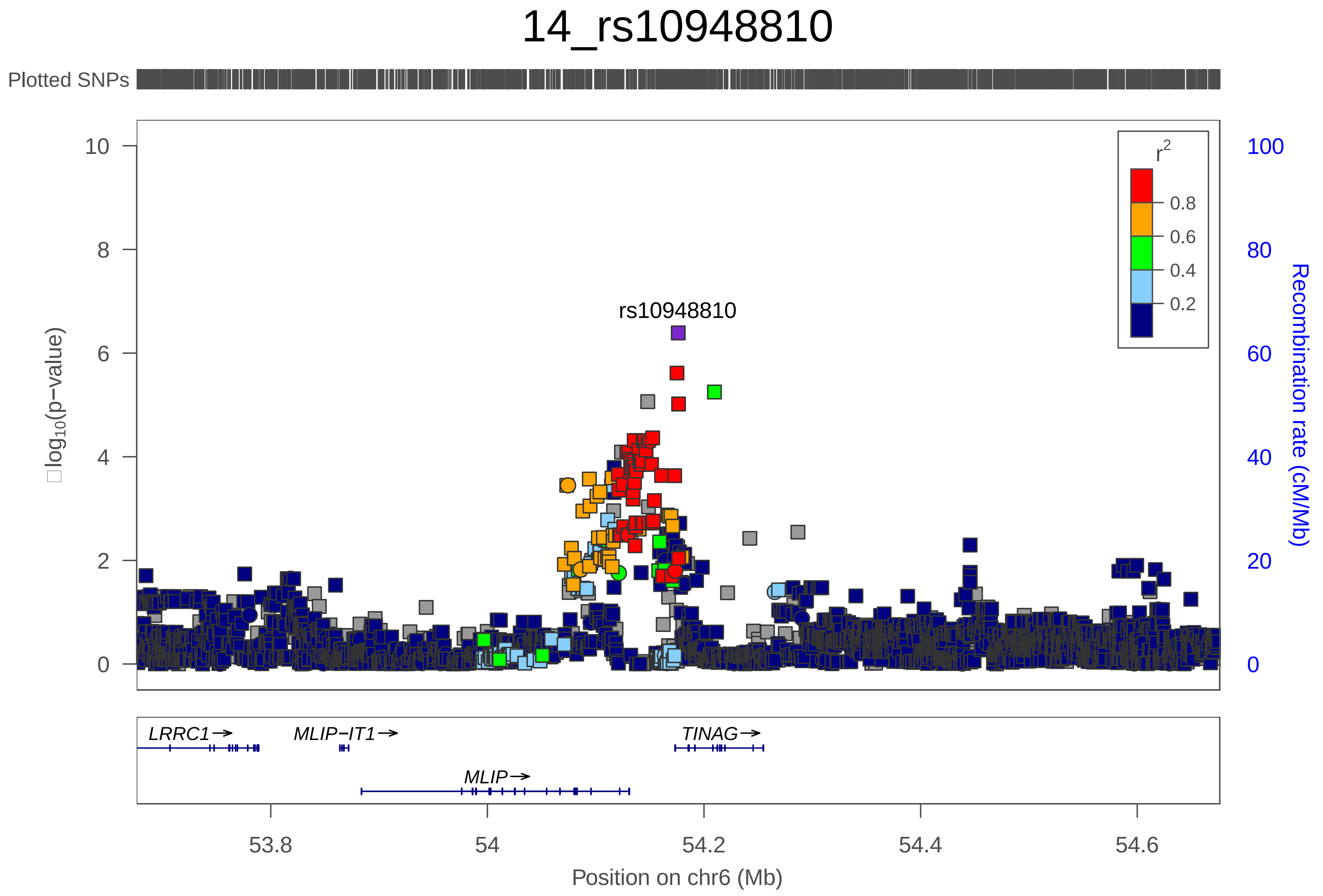

14_rs10948810
Plotted SNPs
rs10948810
r2
0.8
0.6
0.4
0.2
10
8
6
4
2
0
log10(p−value)
100
80
60
40
20
0
Recombination rate (cM/Mb)
LRRC1
MLIP−IT1
TINAG
MLIP
53.8
54
54.2
54.4
54.6
Position on chr6 (Mb)

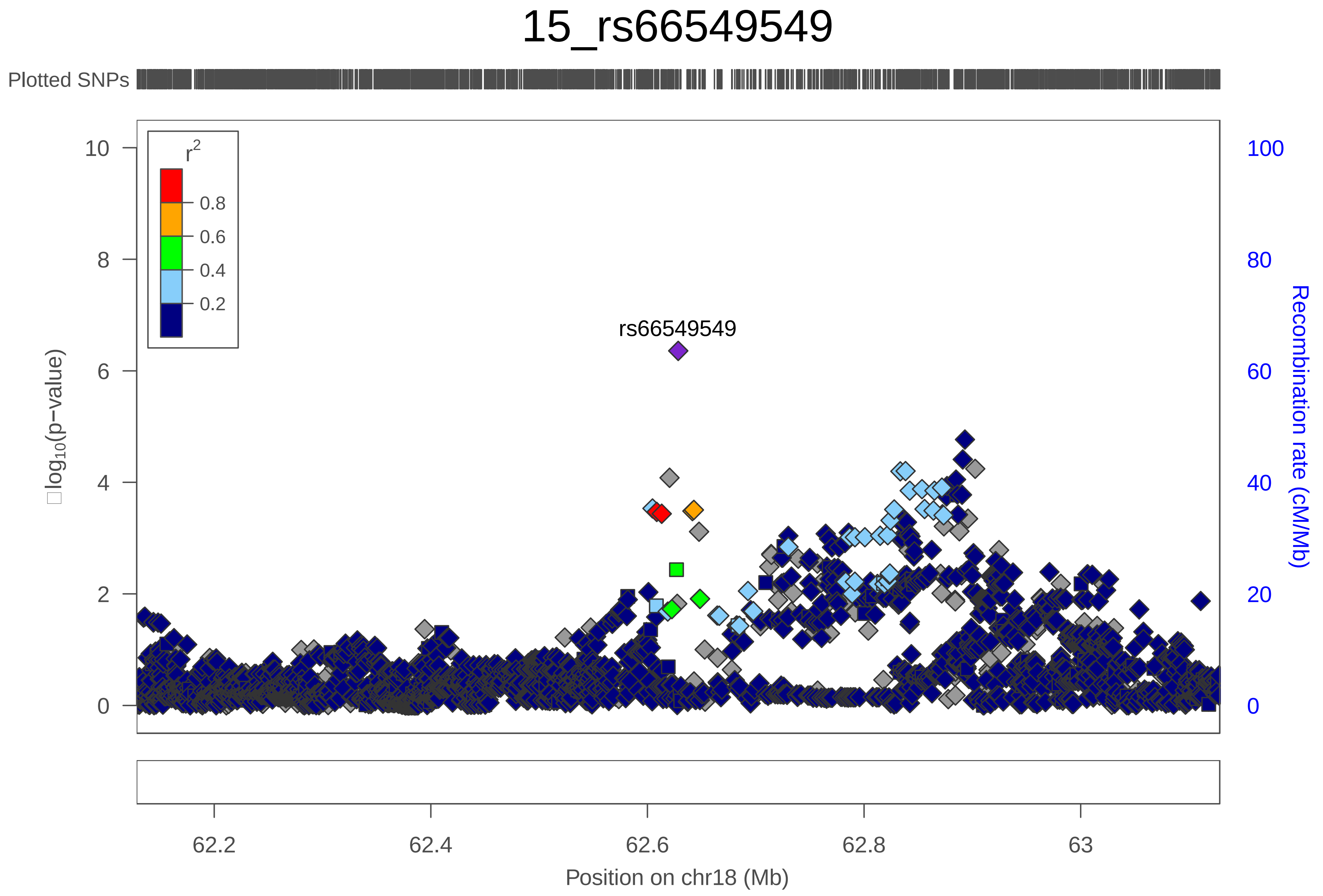

15_rs66549549
Plotted SNPs
r2
0.8
0.6
0.4
0.2
rs66549549
10
8
6
4
2
0
100
80
60
40
20
0
log10(p−value)
Recombination rate (cM/Mb)
62.2
62.4
62.6
62.8
63
Position on chr18 (Mb)

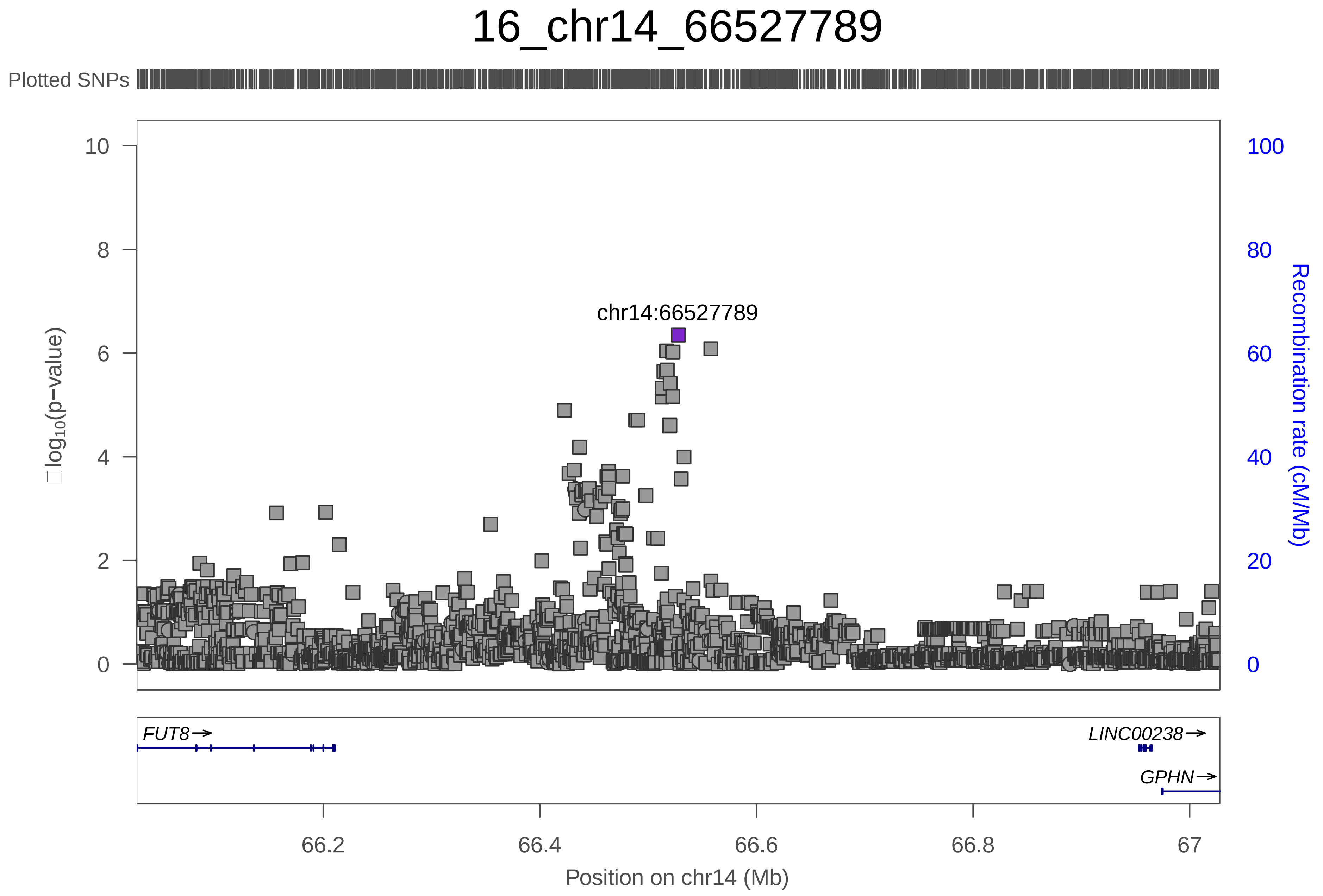

16_chr14_66527789
Plotted SNPs
chr14:66527789
−log10(p−value)
10
8
6
4
2
0
100
80
60
40
20
0
Recombination rate (cM/Mb)
FUT8
LINC00238
GPHN
66.2
66.4
66.6
66.8
67
Position on chr14 (Mb)

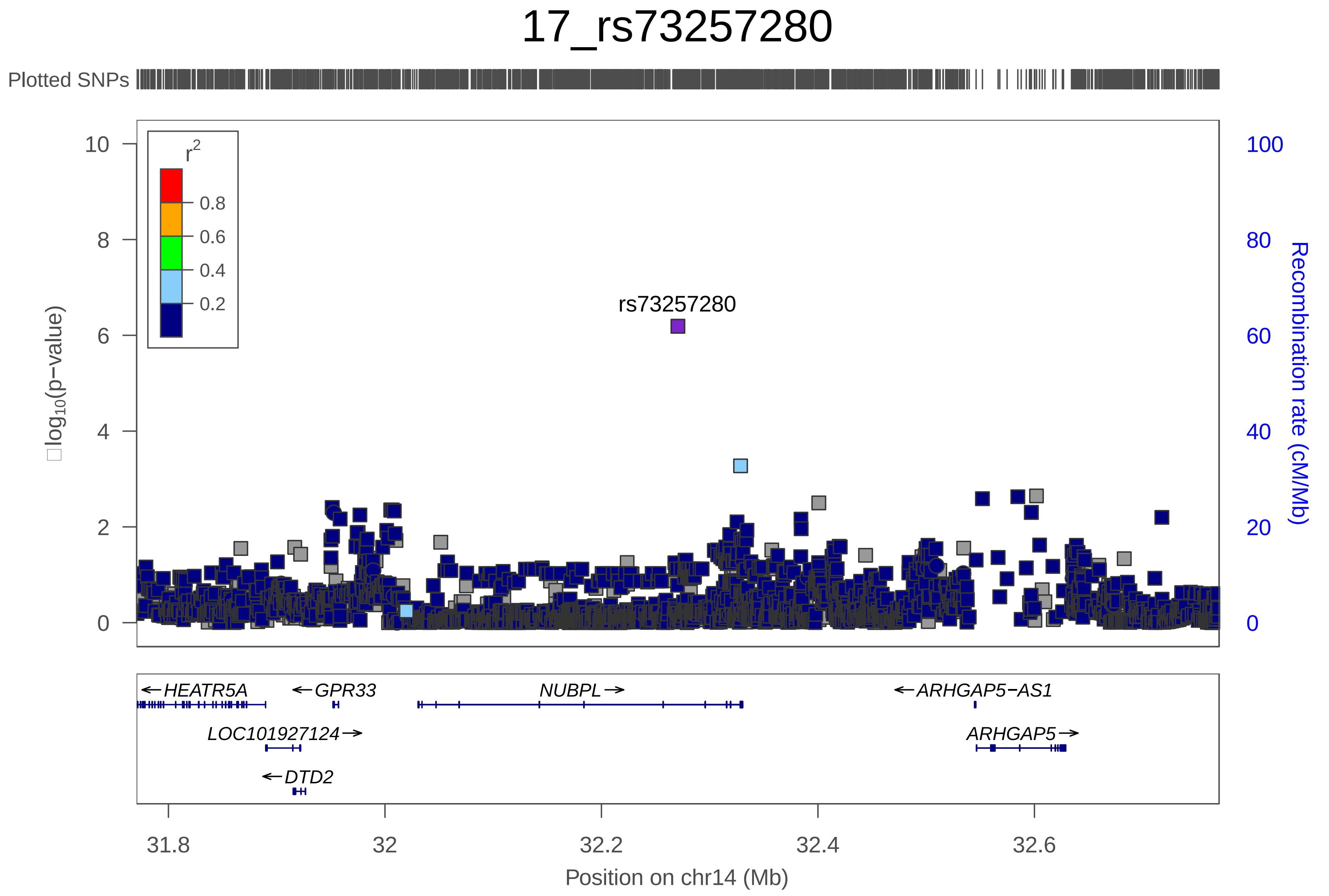

17_rs73257280
Plotted SNPs
r2
0.8
0.6
0.4
0.2
rs73257280
log10(p-value)
Recombination rate (cM/Mb)
10
8
6
4
2
0
100
80
60
40
20
0
HEATR5A
GPR33
NUBPL
ARHGAP5-AS1
LOC101927124
ARHGAP5
DTD2
31.8
32
32.2
32.4
32.6
Position on chr14 (Mb)

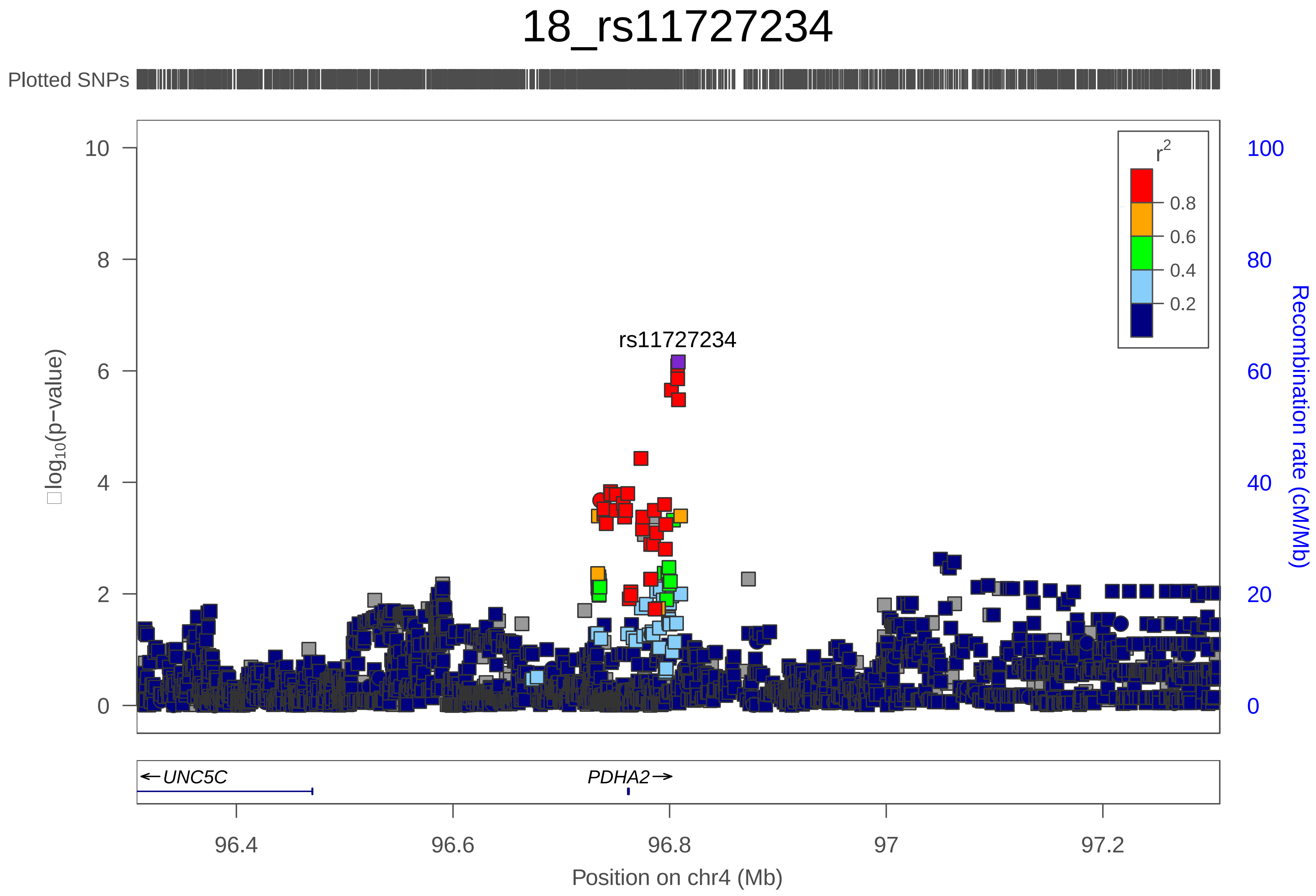
18_rs11727234
Plotted SNPs
rs11727234
10
8
6
4
2
0
−log10(p−value)
100
80
60
40
20
0
Recombination rate (cM/Mb)
r2
0.8
0.6
0.4
0.2
UNC5C
PDHA2
96.4
96.6
96.8
97
97.2
Position on chr4 (Mb)

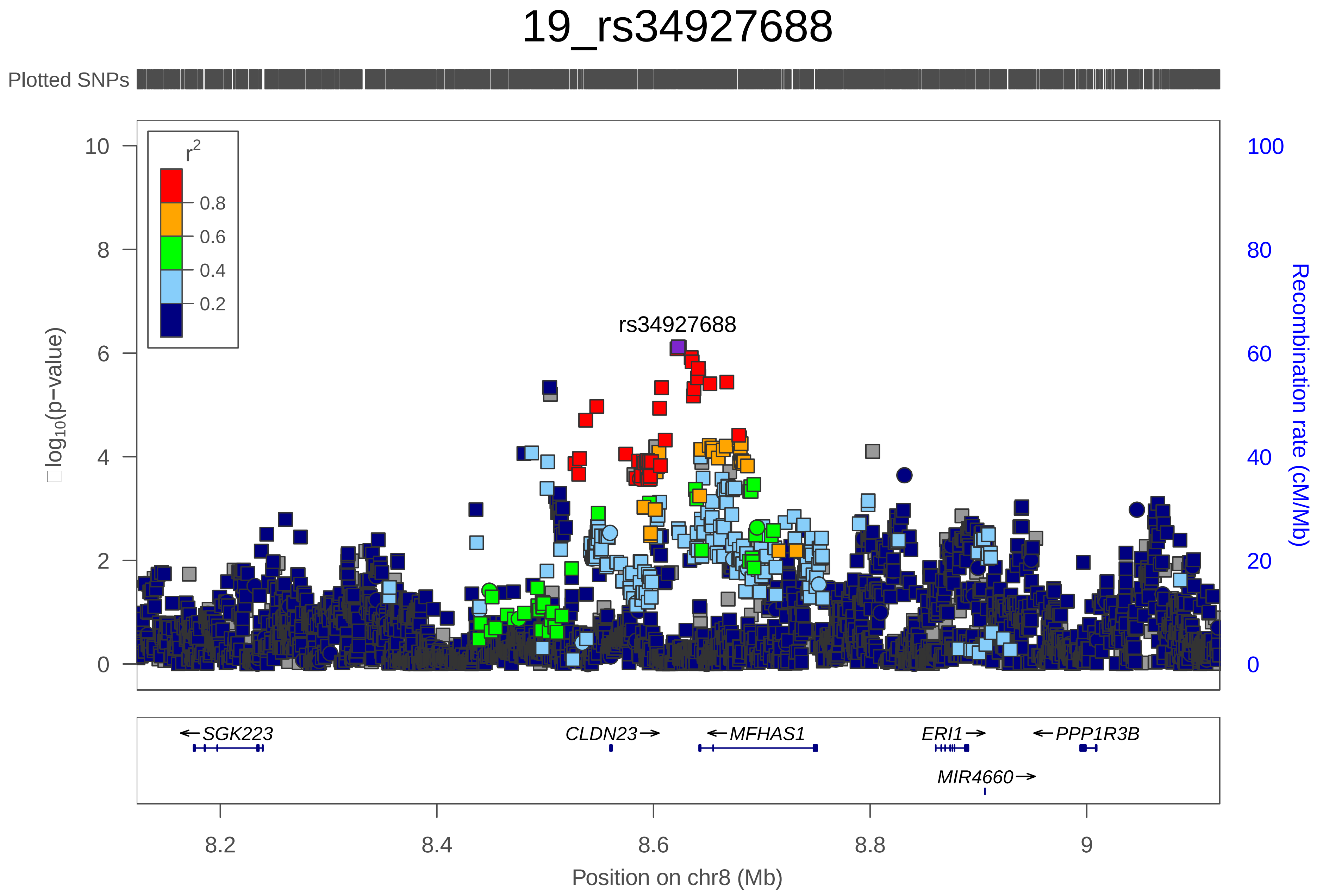
19_rs34927688
Plotted SNPs
r2
0.8
0.6
0.4
0.2
rs34927688
log10(p-value)
Recombination rate (cM/Mb)
10
8
6
4
2
0
100
80
60
40
20
0
SGK223
CLDN23
MFHAS1
ERI1
PPP1R3B
MIR4660
8.2
8.4
8.6
8.8
9
Position on chr8 (Mb)

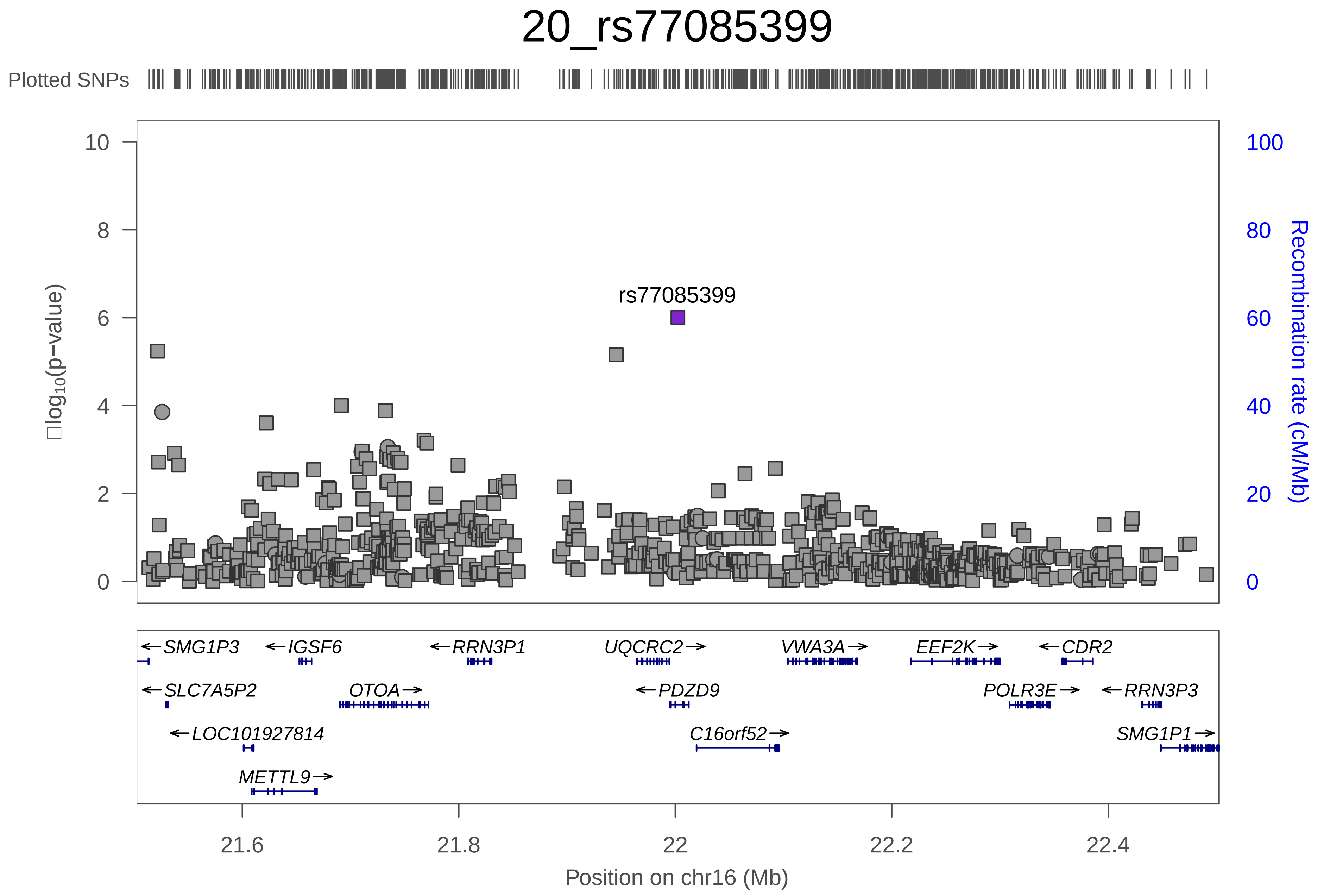

20_rs77085399
Plotted SNPs
-log10(p-value)
Recombination rate (cM/Mb)
rs77085399
SMG1P3
IGSF6
RRN3P1
UQCRC2
VWA3A
EEF2K
CDR2
SLC7A5P2
OTOA
PDZD9
POLR3E
RRN3P3
LOC101927814
C16orf52
SMG1P1
METTL9
21.6
21.8
22
22.2
22.4
Position on chr16 (Mb)
